# Standard CBT versus integrative and multimodal CBT assisted by virtual-reality for generalized anxiety disorder

**DOI:** 10.3389/fpsyg.2022.1008981

**Published:** 2022-09-28

**Authors:** Cosmin Octavian Popa, Florin Alin Sava, Simona Muresan, Alina Schenk, Cristiana Manuela Cojocaru, Lorena Mihaela Muntean, Peter Olah

**Affiliations:** ^1^Department of Ethics and Social Sciences, George Emil Palade University of Medicine, Pharmacy, Science, and Technology of Targu Mures, Targu-Mures, Romania; ^2^Department of Psychology, West University of Timișoara, Timișoara, Romania; ^3^Department of Internal Medicine IV, George Emil Palade University of Medicine, Pharmacy, Science, and Technology of Targu Mures, Targu-Mures, Romania; ^4^Doctoral School of Medicine and Pharmacy, George Emil Palade University of Medicine, Pharmacy, Science, and Technology of Targu Mures, Targu-Mures, Romania; ^5^Department of Psychiatry, George Emil Palade University of Medicine, Pharmacy, Science, and Technology of Targu Mures, Targu-Mures, Romania; ^6^Department of Medical Informatics and Biostatistics, George Emil Palade University of Medicine, Pharmacy, Science, and Technology of Targu Mures, Targu-Mures, Romania

**Keywords:** cognitive-behavioral therapy, generalized anxiety disorder, virtual reality, integrative and multimodal CBT, worries

## Abstract

**Introduction:**

Generalized Anxiety Disorder (GAD) is a prevalent emotional disorder associated with increased dysfunctionality, which has a lasting impact on the individual’s quality of life. Besides medication, Cognitive-Behavioral Therapy (CBT) represents the golden standard psychotherapeutic approach for GAD, integrating multilevel techniques and various delivery formats that enable the development of tailored treatment protocols. The objective of this study was to compare the efficiency of a standard CBT protocol targeting worries, dysfunctional beliefs, and intolerance of uncertainty with an integrative and multimodal CBT intervention augmented with Virtual Reality (VR).

**Materials and methods:**

This study included 66 participants (*M*_age_ = 22.53 years; *SD* = 2.21) with moderate GAD symptoms that were randomized to the standard CBT group (CBTs; *N* = 32) and the Integrative and Multimodal CBT augmented with VR (IM-VRCBT; *N* = 34) group. The interventions comprised 10 weekly sessions conducted by trained CBT therapists, including cognitive restructuring, problem-solving, behavioral exposure, and relaxation techniques. Baseline and post-assessments were conducted with both groups. Primary outcome measures included the Hamilton Anxiety Rating Scale (HARS) and Penn-State Worry Questionnaire (PSWQ) to evaluate the severity of GAD symptoms and worries, respectively. Secondary outcomes involved the administration of Automatic Thoughts Questionnaire (ATQ), Dysfunctional Attitudes Scale (DAS) and Unconditional Self-Acceptance Questionnaire (USAQ).

**Results:**

Both interventions determined statistically significant effects on both primary and secondary outcomes (ps < 0.001) in the expected direction. However, CBTs was associated with higher effect sizes for anxiety (Cohen’s *d* = 2.76) and worries (Cohen’s *d* = 1.85), in contrast to IM-VRCBT. Also, secondary analyses revealed positive correlations between changes in anxiety and worries level and the reduction of dysfunctional cognitive processes.

**Conclusion:**

This research emphasized the effectiveness of CBT interventions for treating adults with moderate GAD symptomatology. Specifically, both interventions were efficient for reducing anxiety symptomatology present at individuals with GAD. However, regarding cognitive dysfunctions like worries, the standard CBT protocol performed better, as compared to the IM-VRCBT. In addition, we conclude that VR could be integrated within CBT interventions in a single protocol for GAD treatment.

## Introduction

From the entire group of anxiety disorders included in the Diagnostic and Statistical Manual of Mental Disorders, fifth edition (DSM-5), the Generalized Anxiety Disorder (GAD) represents one of the most common affective disorders (Penninx et al., 2021). The lifetime and 12-month prevalence of GAD in the general population was estimated at 3.7 and 1.8%, respectively, (Ruscio et al., 2017). Specific symptoms of GAD are uncontrollable worries, excessive anxiety and restlessness, fatigability, irritability, muscle tension, insomnia and concentration difficulties, influencing the individual’s functionality in multiple life roles (American Psychiatric Association, 2013). Moreover, without adequate treatment, GAD presents a tendency toward chronicity (Rynn and Brawman-Mintzer, 2004). Besides these specific symptoms, different cognitive and attentional biases, such as the overestimation of a hypothetic threat or underestimation of one’s capacity to manage stressful situations were described as GAD characteristics (Hirsch et al., 2019). Likewise, attempts to control the occurrence of certain thoughts/thought suppression could represent an important cognitive bias present in GAD (Aydın et al., 2019), as well as other enduring and severe psychopathologies (Nirestean et al., 2012, 2016; Popa et al., 2020). Therefore, individuals use various safety-seeking strategies, which reinforce dysfunctional attitudes and biased perceptions of personal coping abilities, contributing to a raise of uncertainties and self-doubts (Gústavsson et al., 2021). In addition, cognitive distortions, like catastrophizing and emotional reasoning, along with unproductive behavioral strategies, such as procrastination, often occur in GAD (Mac Leod and Rutherford, 2004; Newman et al., 2017). In this way, avoidance or overcompensation coping styles that are often associated with anxiety in GAD, along with a variety of psychological disorders, may impact overt behavior in complex ways (Nirestean et al., 2014).

From the perspective of treatment, many studies showed that, besides medication, Cognitive-Behavioral Therapy (CBT) constitutes one of the most efficient psychological interventions for GAD (Dugas et al., 2010; Cuijpers et al., 2014). In this regard, both forms of treatment are considered effective (Bandelow et al., 2008), and the antidepressant and/or anxiolytic medication can be successfully augmented with different CBT orientations (Veale and Stout, 2010; Orvati Aziz et al., 2020). According to the existing empirical evidence, CBT approaches are flexible and highly adaptable psychological interventions that knew a rapid development towards an integrative orientation that, beyond the traditional delivery, also embraced digital, Internet-based and VR-augmented programs (Lindner, 2021). In this context, CBT can be used like a unique intervention, or it can be assisted/augmented with Virtual Reality (VRCBT) (Cardoş et al., 2017; Carl et al., 2019).

There are many CBT protocols in the treatment of GAD, including diverse strategies with the aim of decreasing the level of anxiety symptoms and associated comorbidities, as well as dysfunctional behaviors (Ballenger et al., 1998; Borkovec and Ruscio, 2001). Among these, the psychological protocols developed by David et al. (2010); Beck (2011, 2021); Robichaud (2013), and Robichaud et al. (2019) may constitute, the psychological treatment cornerstones in GAD. In this way, according to Beck and Haigh (2014), the cognitive model considers the pathological anxiety a product of overestimating internal or external threats (Beck and Haigh, 2014). The therapeutic process in this approach includes cognitive debating and restructuring of dysfunctional beliefs and negative automatic thoughts (NATs), behavioral exposure, relaxation techniques and modification of dysfunctional coping mechanisms (Beck et al., 2005). Also, Robichaud (2013) proposed a new CBT approach for treating GAD, targeting intolerance of uncertainty (CBT-IU) in particular. This treatment is focused on the recognition and evaluation of worries, identification of uncertainty, application of different cognitive and behavioral exposure scripts, cognitive restructuring of positive beliefs about worries, accompanied by, importantly, the development of new behavioral skills using problem-solving. Hence, the CBT-IU protocol in GAD addresses both dysfunctional cognitive processes and specific symptoms (Robichaud, 2013). Besides, metacognitive processes can be present in GAD (Wells, 1995), some authors showing that the cognitive restructuring of NATs and their meaning may be considered a therapeutic intervention at a metacognitive level (Moritz and Lysaker, 2019; Popa et al., 2022). Furthermore, given the high frequency of comorbidities associated with GAD (American Psychiatric Association, 2013), an integrative and multimodal CBT (IM-CBT) approach allows a tailored psychotherapeutic process for patients targeting multiple functioning areas by incorporating techniques from classical and updated cognitive and behavioral perspectives (David et al., 2010; Fodor et al., 2018). The IM-CBT was built on the ABC cognitive model derived from Rational Emotive Behavioral Therapy (REBT) (David et al., 2010) that concentrates on changing irrational beliefs (B) at the multilevel of cognitions (cold and hot cognitions), along with emotional regulation techniques, resulting in the reduction of unhealthy emotions and behaviors (C) linked to activating events (A) (David and Szentagotai, 2006). The transition from the classical CBT approach (the “second-wave”) aiming symptoms reduction to the “third-wave” orientations was motivated by the need to refine cognitive and behavioral techniques, considering the importance of context versus content. There are recent orientations that incorporate both “second- and third-wave” techniques in an integrative and multimodal intervention. Therefore, integrative CBT approaches address psychopathology by shifting the focus from a pure categorical towards a dimensional perspective pursuing the identification of dysfunctional processes behind psychopathology (Hayes and Hofmann, 2017). Given the recent advancement of technology applications in the health field, the digitalization tendency could facilitate the psychotherapeutic process (Gregg and Tarrier, 2007).

Specifically, possible advantages of augmenting psychological interventions with Virtual Reality (VR) technology may include the reduction of both financial and temporal costs for individuals suffering from mental health disorders, as well as the opportunity to generate scenarios that are not possible in real world (Freeman et al., 2017). Also, we hypothesize that using VR can be a helpful tool for early career psychotherapists by ensuring a standardized application of challenging strategies (e.g., exposure to anxiety-inducing scenarios) and minimizing interferences like the practitioner’s emotions or limited professional experience. In addition to standard psychological interventions, Virtual Reality technology was predominantly applied in anxiety disorders, either as stand-alone strategies or in combination with other forms of psychotherapy (Fodor et al., 2018; Anderson and Molloy, 2020; Wu et al., 2021). Regarding GAD, VR was applied to counteract physiological reactions of anxiety using relaxation scenarios accompanied by audio narrative support, but also to expose the individual to stressful or potentially catastrophic standardized scenarios developed to trigger common uncertainties and worries (Gorini et al., 2010; Repetto et al., 2013; Guitard et al., 2019). The exacerbation of anxiety levels during the immersion could promote VR as an alternative to the classic worst-case scenario exposure in GAD treatment (Guitard et al., 2019). To our knowledge, previous studies compared different VR protocols for GAD with waiting lists and other specific exposure (Guitard et al., 2019) or relaxation techniques (Gorini et al., 2010; Repetto et al., 2013), without using standard or VR-augmented CBT interventions.

Thus, the general objective of our study is to compare the effectiveness of two CBT protocols for alleviating GAD symptoms. First, standard CBT based on the Beck protocol (Beck, 2011, 2021), focusing on GAD and associated comorbidities, was combined with the Robichaud protocol (Robichaud et al., 2019), addressing worries, uncertainty, problem solving and behavioral exposure, also integrating mindfulness and relaxation techniques. Second, since VRCBT protocols were implemented in relation to other anxiety disorders (Maples-Keller et al., 2017; Fodor et al., 2018), the IM-VRCBT applied in our study involved cognitive restructuring of irrational beliefs, problem-solving methods, besides the VR technology for exposure to anxiety-inducing and relaxation scenarios. The specific objective of our research was to identify alternative interventions in GAD, starting from prominent techniques used in IM-CBT and VRCBT.

## Materials and methods

### Ethical approval

This clinical trial was approved by the Ethics Committee of George Emil Palade University of Medicine, Pharmacy, Sciences and Technology of Targu Mures, under the approval number 1138 from 22th Sep 2020, as part of a research grant won within a research funding competition.

### Participants

Participants were recruited from the George Emil Palade University of Medicine, Pharmacy, Sciences and Technology of Targu Mures, Faculty of Medicine. In the first phase of the study, a screening regarding the presence/absence of clinical anxiety was conducted. From the total number of 1920 individuals who went through the screening, 93 individuals were selected, presenting a score over 25 points at the Leahy Anxiety Checklist, which was considered a cut-off point for moderate anxiety (Leahy et al., 2011). In addition, the Structured Clinical Interview for DSM-5 (SCID-5-Clinical Version) was used (First et al., 2016) for the assessment of Generalized Anxiety Disorder (GAD) and Major Depressive Disorder diagnosis criteria. For both groups the inclusion criteria were: (1) the GAD diagnosis according to DSM-5 criteria; (2) the absence of psychiatric medication. The exclusion criteria were: (1) the presence of psychotic symptoms or the schizophrenia diagnosis; (2) severe personality disorders. From the participants that were initially selected, 16 were excluded because the inclusion criteria were no longer met or refused to attend further in the study. Over the course of the research, drop-out occurred for 13 individuals. Therefore, 66 individuals who fulfilled the diagnosis criteria for GAD were enrolled in the entire intervention process. The mean age for these 66 participants included in the analysis is 22.53 years (*SD* = 2.21), most participants being females (78.8%). There were no age or gender significant differences between the two treatment groups.

### Measures

#### Primary outcome measures

The Hamilton Rating Scale for Anxiety (HRSA; Hamilton, 1959) is an interview for the measurement of anxiety severity, comprising the following 14 symptom components: anxious mood, tension, fears, insomnia, intellect/ cognition, depressed mood, somatic (muscular, sensory, cardiovascular, respiratory, gastro-intestinal, genito-urinary, and autonomic) symptoms, and behavioral observations at interview. Each item is rated by clinicians using a 5-point Likert scale (from 0 = none to 4 = very severe). Final scores are calculated by adding the individual rating for each item, higher scores indicating increased anxiety severity. A score over 20 is considered the cut-off, indicating a clinical intensity of anxiety. Regarding the psychometric properties of the scale, the weighted correlations mean between raters was 0.89 following the z transformation, which reflects a high reliability of the instrument, as assessed following the application on an outpatient sample (Hamilton, 1959). Also, HRSA demonstrated good internal consistency, indicating an alpha coefficient of 0.89 (Kummer et al., 2010). The instrument was adapted and proved to be reliable for the Romanian population, as indicated by the obtained inter-rater concordance coefficient of 0.84 (Hamilton, 2007).

The Penn State Worry Questionnaire (PSWQ; Meyer et al., 1990) is a 16-item self-report scale that measures the tendency to worry frequently, covering common cognitive features that accompany generalized anxiety. Respondents are asked to rate each answer on a 5-point Likert scale (from 1 = not at all typical of me to 5 = very typical of me). For the majority of items, the raw score is considered (for example, “My worries overwhelm me” or “Once I start worrying, I cannot stop”), while some items are inversely scored (for example, “I do not tend to worry about things” or “I find it easy to dismiss worrisome thoughts”). The final score is calculated by summing up all the responses. The instrument demonstrated high internal consistency, with an alpha coefficient of 0.93, as well as very good test–retest reliability, with r = 0.92, in one of the initial validation studies that included both non-clinical, as well as clinical samples (Meyer et al., 1990). The PSWQ was previously used with a Romanian cohort from the general population, indicating good internal consistency, with an alpha coefficient of 0.80 (Pasarelu et al., 2015).

#### Secondary outcome measures

The Automatic Thoughts Questionnaire (ATQ; Hollon and Kendall, 1980) is a self-report questionnaire that evaluates the frequency of negative thoughts regarding the self, others, and the world, capturing the typical cognitive content related to emotional difficulties. The ATQ includes statements like “I’m no good” and “What’s the matter with me?.” Responses are rated on a 5-point Likert scale from 1 = not at all to 5 = all the time. Total score is calculated by adding the score for each item. The shortened version of the scale consisting of 15 items was used in this study. The questionnaire proved to have excellent internal consistency, with an alpha coefficient of 0.96 for the 15-item version (Netemeyer et al., 2002). The validation study of the Romanian version indicated very good reliability, with an alpha coefficient of 0.92 on an adult sample from the general population (Hollon and Kendall, 2007).

The Dysfunctional Attitudes Scale (DAS; Weissman and Beck, 1978) is a 40-item self-report measure of maladaptive beliefs and cognitive distortions that correlate with the occurrence of psychopathology. The instrument has two parallel forms (A and B), responses being quantified on a 7-point Likert scale from 1 = totally disagree to 7 = totally agree. The A form was implemented in this study, consisting of assumptions like “If I do not do as well as other people, it means I am an inferior human being” or “My value as a person depends greatly on what others think of me.” Final scores are calculated by adding individual ratings, considering that several items are inversely scored (for example, “Making mistakes is fine because I can learn from them”). From the perspective of psychometric properties, the instrument is highly reliable, with an alpha coefficient of 0.86 after the application in an adult population of university graduates (Weissman and Beck, 1978). The questionnaire has been adapted to be used on an adult sample in Romania, resulting in equivalent reliability, with an alpha coefficient of 0.86 (Macavei, 2006).

The Unconditional Self-Acceptance Questionnaire (USAQ; Chamberlain and Haaga, 2001) is a 20-item self-report scale that evaluates the degree of self-acceptance, or to which a person uses rational or rather irrational beliefs for self-assessment. A 7-point Likert scale from 1 = almost always untrue to 7 = almost always true is used for rating the answers. The instrument is based on the rational-emotive behavior theory of Albert Ellis, including some items like: “When I receive negative feedback‚ I take it as an opportunity to improve my behaviour or performance,” “I avoid comparing myself to others to decide if I am a worthwhile person.” The sum of all individual items is counted for the final score, which includes reverse-scored items (e.g., “When I am criticized or when I fail at something‚ I feel worse about myself as a person”). In the original validation study, the obtained alpha coefficient was 0.86, reflecting high internal consistency (Chamberlain and Haaga, 2007). The instrument was adapted for Romanian population on an adult sample, demonstrating good internal consistency, with an obtained alpha coefficient of 0.73 (Chamberlain and Haaga, 2007).

### Procedure

Initially, participants were recruited through an online form that provided information regarding the purposes and methods of this research. After the application of inclusion and exclusion criteria, eligible participants met a research team member to discuss general characteristics about the intervention process and signed the written consent, ensuring confidentiality and protection of data. During the same initial meeting, the initial assessment was carried out by two research team members. After the intervention, the final assessment was conducted by each therapist involved in the study.

**Figure 1:**
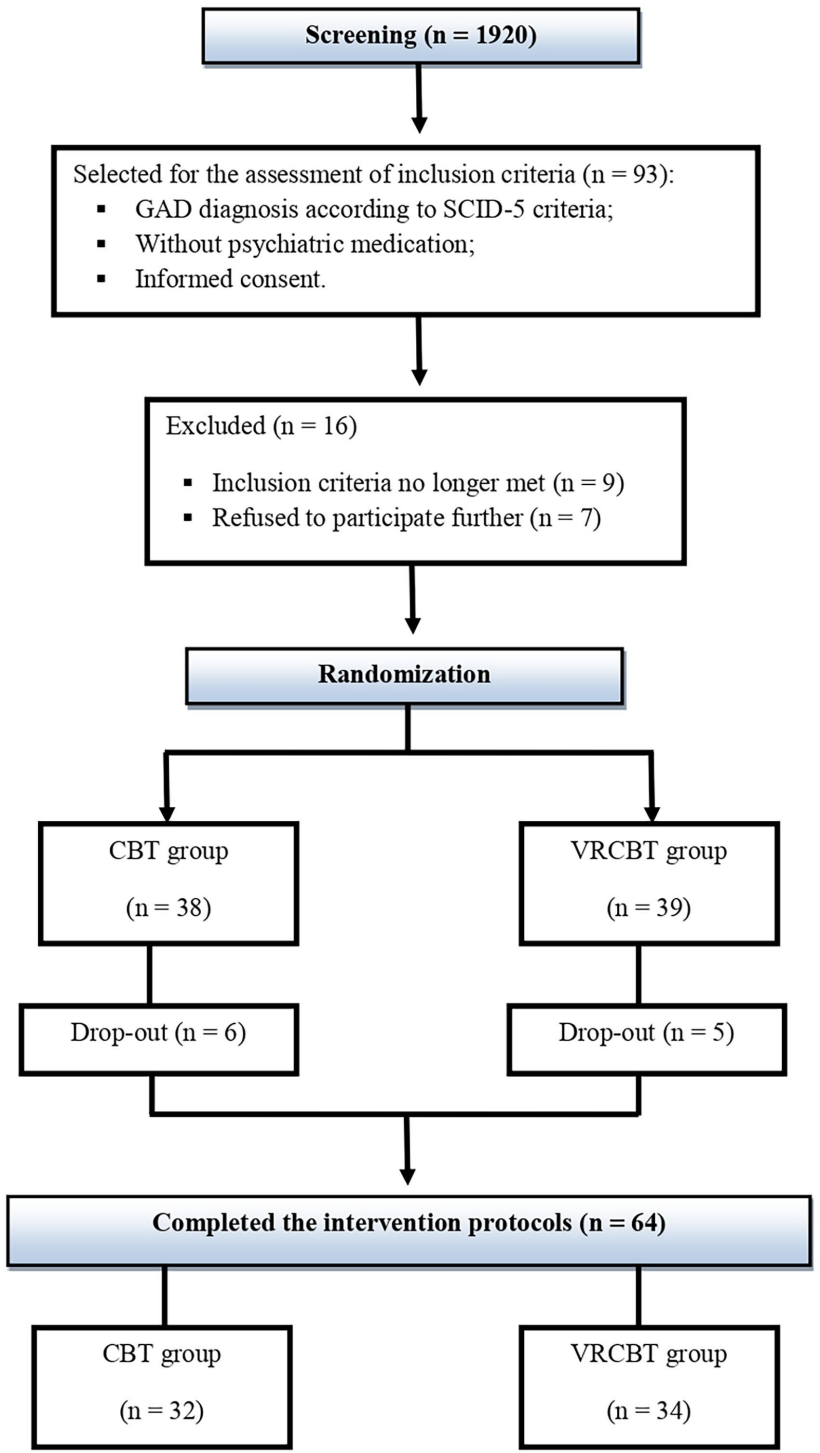
Flow diagram of main study procedures and randomization process.

The sampling process involved a simple randomization method, using ID numbers for dividing participants into the standard CBT group (CBTs), including 32 participants (25 females, 7 males; *M*_age_ = 22.75; *SD* = 2) that received a traditional CBT intervention, and the IM-VRCBT group (IM-VRCBT) including 34 participants (27 females, 7 males; *M*_age_ = 22.14; *SD* = 1.95) that received an IM-CBT intervention augmented with VR (see Figure 1).

A clinical assessment was conducted for both groups, before and after the interventions. The specific CBT intervention used in the CBTs group was based on the treatment plan for GAD elaborated by Robichaud et al. (2019) completed with the model derived from the Beck (2011) cognitive therapy. The cognitive model for case conceptualization was selected to provide the structure of the intervention (Beck, 2011, 2021). The CBTs intervention consisted in a total number of 10 weekly sessions with a duration of 60 min, delivered within 10 weeks. Various CBT techniques were implemented, including: (a) cognitive reappraisal; (b) approaching uncertainties and worries; (c) problem solving; (d) behavioral exposure; (e) relaxation techniques; and (f) mindfulness. More specific, sessions 1–2 were focused on the clinical case conceptualization and psychoeducation, along with the application of different methods for identifying and evaluating worries, uncertainty and dysfunctional beliefs, as well as cognitive restructuring of NATs. Sessions 3–4 emphasized the relation between uncertainty and worries, followed by the application of cognitive restructuring techniques, combined with specific problem-solving strategies. Session 5 was allocated to the beginning of behavioral exposure, built on the worst-case scenario, first in written and subsequently in imagery (*in-vitro* exposure). Autogenic training (Schultz and Luthe, 1959) was used in session 6 as a relaxation technique. During sessions 7, mindfulness techniques focused on breathing and bodily sensations (Gordon and Borushok, 2017) were applied. Session 8 was reserved for behavioral exposure to uncertainty, while session 9 concentrated on mindfulness. Cognitive restructuring continued each time after the implementation of exposure or relaxation strategies. Session 10 was dedicated to progress maintenance and preventing relapses. Between sessions, different homework assignments were prescribed, such as: *in-vivo* behavioral exposure, behavioral experiments and exposure to uncertain situations in everyday life.

For the IM-VRCBT group, an integrative and multimodal CBT approach that derives from David and Freeman (2015) was applied (David and Freeman, 2015; David and Cristea, 2018) augmented with Virtual Reality (VR) techniques. The intervention consisted, in a total number of 10 weekly sessions, with the same duration and delivery time as for the CBTs group. This protocol also included: (a) irrational beliefs restructuring; (b) cold and hot cognitions identification, evaluation and debate; (c) problem solving; (d) classic and VR-based exposure; (d) VR relaxation techniques; and (e) VR mindfulness. The model included: (1) the ABC model (David et al., 2010) for the clinical case conceptualization; (2) debating irrational beliefs; (3) modifying cold and hot cognitions; (4) decreasing low frustration tolerance (LFT); and (5) generating alternative rational attitudes. This intervention was augmented with VR techniques starting from session 5 for exposure, relaxation techniques and mindfulness. For all VR-based strategies, a non-immersive setting based on the Oculus Rift software was implemented. Therefore, in sessions 1–2, the ABC model was presented, applying specific techniques for irrational beliefs restructuring. This approach was continued in sessions 3–4, when worries and uncertainty were addressed by modifying hot cognitions and decreasing the level of LFT. During session 5, the written worst case scenario exposure was conducted, followed by the VR exposure using the “Richie’s Plank Experience” application (Toast VR, 2019). In sessions 6 and 7, the “Guided Meditation VR” application (Cubicle Ninjas, 2016) was used for relaxation and mindfulness techniques, respectively. In session 8, the “Face your Fears” VR application (Oculus Studios, 2016) was used for exposure to uncertainty. In session 9, mindfulness techniques were resumed, using the “Guided Meditation VR” application. Also, cognitive restructuring was carried over during the entire intervention. The last session was focused on progress consolidation and relapse prevention. Throughout the treatment, different homework assignments were established, including exposure to anxious stimuli, exposure to uncertainty and behavioral experiments.

#### Standard CBT versus IM-CBT intervention augmented with VR

The CBTs and IM-CBT were constructed based on the same CBT model/framework focusing on specificy psychopathological symptoms and working with dysfunctional beliefs, problem solving and skills development (Alford and Beck, 1997). For this reason, there are just a few disparities between the structure of these protocols, consisting in the use of basic “third-wave” techniques within the IM-CBT. First, CBTs based on the classical cognitive model described above, directly targeted NATs, dysfunctional beliefs, intolerance of uncertainty and worries, using different cognitive restructuring techniques. On the other hand, the IM-CBT combined the classical cognitive model with different processes/methods from the “third-wave” orientations (David and Cristea, 2018). Therefore, the main differences between these two approaches consisted in the cognitive case conceptualization, and the way in which various CBT treatment techniques were applied/combined. Second, while classic exposure scenarios, built on the worse-case scenario of each participant, were applied in both treatments, standardized VR scripts replacing classical behavioral exposure were used within the IM-CBT. Third, the CBTs implemented established relaxation and mindfulness procedures, providing a higher involvement of therapists in the process, whereas the IM-CBT applied exclusively VR-based techniques as substitutes for the classical face-to-face strategies.

### Therapists

A total of 20 CBT psychotherapists were implicated in this study, from this number 7 being trained as clinical psychologists and 13 as psychiatrists. Out of the total number, 6 psychotherapists were assigned to work with the CBTs group, while 9 worked with the IM-VRCBT group exclusively. The remaining 5 psychotherapists provided interventions within both groups, that were the most experienced from the entire group of therapists, as required for synchronizing treatment progress among both groups. Regarding the professional background, all involved psychotherapists had at least 1 year of CBT experience either within the public health system (hospitals, non-governmental organizations, institutions providing clinical psychological services), or private practice. Psychotherapists working with the CBTs group had an average clinical experience of 6.12 years (*SD* = 4.05), whereas psychotherapists working with the IM-VRCBT group had an average clinical experience of 4.14 years (*SD* = 4.76). Before applying the interventions, all involved therapists were trained in the specific protocols implemented in this research.

## Results

### Intervention effects on primary outcomes

**Figure 2:**
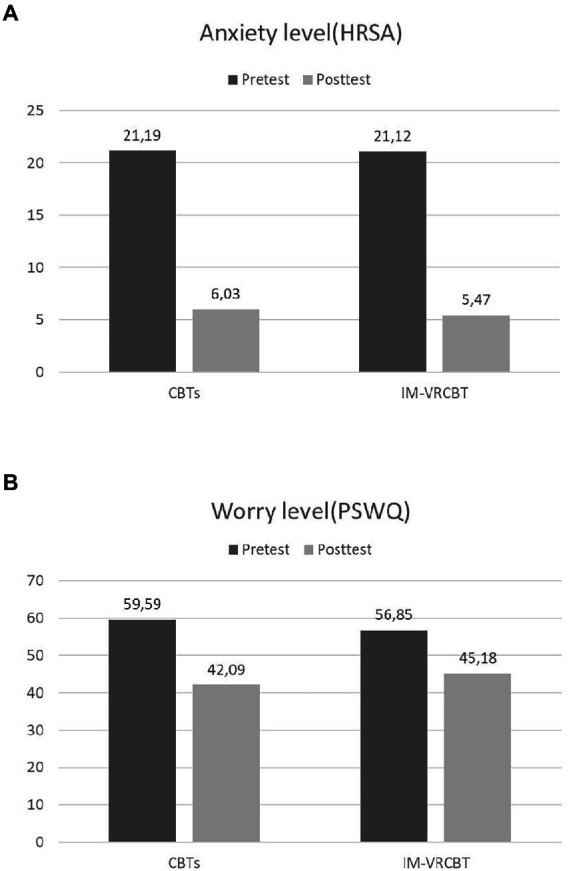
Differences in anxiety level, as measured with the Hamilton Rating Scale for Anxiety **(A)** and worry level, as measured with the Penn-State Worry Questionnaire **(B)**, between the CBTs and the IM-VRCBT groups from pre- to posttest.

The 2 (Treatment Condition) × 2 (Time) mixed-model ANOVA revealed no significant treatment by time interaction on HRSA–*F* (1, 64) = 0.06, *p* = 0.803, η^2^ = 0.001, but it revealed a significant treatment by time interaction on PSWQ–*F*(1, 64) = 5.13, *p* = 0.027, η^2^ = 0.074. Likewise, there were significant main effects for Time (*ps* < 0.001). There were no statistically significant differences between the interventions from pre- to posttest. Hence, whereas both interventions were effective in reducing the anxiety and worry levels (*ps <* 0.001), the CBTs was slightly more effective than IM-VRCBT in reducing the accompanying worries, and both were equally effective in reducing the anxiety level. Higher pretest – posttest effect sizes were found for anxiety (Cohen’s *d* of 2.76 for the CBTs group and of 2.34 for the IM-VRCBT group) than for worry (Cohen’s *d* of 1.85 for the CBTs group and of 0.97 for the IM-VRCBT), but all values indicate strong intervention effects (see Figure 2).

### Intervention effects on secondary outcomes

The 2 (Treatment Condition) × 2 (Time) mixed-model ANOVA revealed no significant treatment by time interaction (all *ps* > 0.05) for any of the secondary outcomes. In all cases we did find significant differences for the within-group comparisons (all *ps* < 0.001) in the expected direction (see Table 1). The pretest – posttest effect sizes were Cohen’s *d* of 1.51 for ATQ, 1.44 for DAS and 1.79 for USAQ in the CBTs condition, and of 1.11 for ATQ, 0.90 for DAS and 1.101 for USAQ in the IM-VRCBT condition, respectively. All values indicate strong intervention effects.

**Table 1:** Mean (SD) for the participants in the two groups and the entire sample included in analyses.

**Variable**	**CBTs intervention (*n* = 32)**	**IM-VRCBT intervention (*n* = 34)**	**All participants (*n* = 66)**	**Cohen’s *d* (CBTs – IM-VRCBT)**
HRSA				
Baseline	21.19 (6.28)	21.12 (7.89)	21.15 (7.10)	−0.01
Posttest	6.03 (4.32)	5.47 (5.13)	5.74 (4.73)	−0.12
PSWQ				
Baseline	59.59 (8.31)	56.85 (11.64)	58.18 (10.18)	−0.27
Posttest	42.09 (10.31)	45.18 (12.33)	43.68 (11.41)	0.27
ATQ				
Baseline	43.63 (11.55)	43.26 (13.99)	43.44 (12.77)	−0.03
Posttest	27.56 (9.03)	29.06 (9.96)	28.33 (9.48)	0.16
DAS				
Baseline	151.87 (30.89)	139.29 (43.61)	145.39 (38.22)	−0.33
Posttest	106.37 (32.17)	103.35 (29.32)	104.82 (30.53)	−0.10
USAQ				
Baseline	69.34 (12.53)	73.21 (18.59)	71.33 (15.94)	−0.24
Posttest	94.19 (14.95)	91.62 (17.60)	92.86 (16.29)	−0.15

HRSA, Hamilton Rating Scale for Anxiety; PSWQ, Penn State Worry Questionnaire; ATQ, Automatic Thoughts Questionnaire; DAS, Dysfunctional Attitudes Scales; USAQ, Unconditional Self-Acceptance Questionnaire.

### Other secondary analyses

Theory suggests that CBT efficacy in reducing anxiety and worry is associated with debating and restructuring the NATs (Clark and Beck, 2010a,b). We included two measures of dysfunctional thoughts – ATQ and DAS. Each of them allows us to explore whether there is a significant correlation between the magnitude of change in reducing such dysfunctional / automatic beliefs and the magnitude of change in the level of primary outcomes (anxiety and worry). The results support this connection, particularly for changes in worries. Thus, there was a positive association between the magnitude of decrease in worrying and the magnitude of decrease in dysfunctional beliefs–*r* (64) = 0.55, *p* < 0.001, and the magnitude of decrease in negative automatic thoughts, respectively–*r* (64) = 0.65, *p* < 0.001. Likewise, there was a positive association between the magnitude of decrease in anxiety and the magnitude of decrease in negative automatic thoughts–*r* (64) = 0.30, *p* = 0.014, and a non-significant association between magnitude of change in anxiety and magnitude of change in dysfunctional thoughts–*r* (64) = 0.02, *p* = 0.88. A similar pattern of results was found when investigating unconditional self-acceptance changes. No association between the change scores in USAQ and anxiety was found–*r* (64) = 0.02, *p* = 0.83, and a strong negative association between the change score in USAQ and change score in PSWQ was found–*r* (64) = −0.64, *p* < 0.001. These results rely on correlation analyses, therefore causality inferences are not supported.

## Discussion

The aim of the present study was to compare the effectiveness of a standard CBT and an IM-VRCBT protocol for adults with moderate GAD, showing that both interventions are equivalently successful in reducing the intensity of anxious manifestations and dysfunctional cognitive processes involved in this disorder. Several investigations found that CBT is the golden standard psychological treatment in GAD (Ladouceur et al., 2000; Simon et al., 2021). Other active treatments, like biofeedback (Biswas and Chattopadhyay, 2001), applied relaxation (Arntz, 2003) and different psychological interventions (Koszycki et al., 2010) proved to be less effective than CBT. In the same way, our outcome is in line with previous research indicating that brief CBT treatments are associated with a considerable decline of GAD symptoms over a relatively short interval (Xie et al., 2019; LeBlanc et al., 2021).

From the perspective of primary outcomes, we demonstrated a significant reduction of anxiety symptoms and worries after both treatment protocols. As reflected in our results, high remission rates of anxiety symptoms in GAD were outlined following CBT interventions (Borkovec and Costello, 1993; Kim et al., 2009). However, as compared to the IM-VRCBT, the CBTs presented a higher improvement related to the cognitive dysfunctional processes of GAD, like worries (Meyer et al., 1990). Previous evidence underlined the effectiveness of cognitive interventions focused both on symptoms and cognitive dysfunctional processes present in GAD, like worries (Covin et al., 2008; Hirsch et al., 2019; Miller et al., 2021). Ideally, either of these CBT based interventions (CBTs or IM-CBT) would benefit to a higher extend from a simple comparison between a given treatment and the VR enhanced version of itself. However, in line with previous studies, we assume that our outcome regarding the increased efficiency of CBTs for reducing the worries level could be attributed to the face-to-face protocol, compared to the IM-CBT with VR enhancement (Fodor et al., 2018), which was a mixed intervention. Hence, future investigations should explore the mediating/changing processes involved in the face-to-face CBT protocol and the mixed approach involving VR, respectively. Moreover, a comparison between CBTs and IM-VRCBT is useful from a pragmatic clinical perspective, taking in account that VR could substitute the face-to-face behavioral exposure, mindfulness and relaxation techniques, focusing on reducing anxiety symptoms in GAD.

Furthermore, our results emphasized that psychological interventions built on a CBT conceptualization are successful in terms of reducing both symptom and cognitive dysfunctional process outcomes. This idea was previously supported by Stefan et al. (2019) in a comparative analysis of three different CBT approaches in adults with GAD, concluding that these protocols are equally efficient for diminishing anxiety symptomatology, uncertainty, worries and NATs, given the strong correlations observed between these concepts. Particularly, a significant decrease of anxiety and worries was obtained, the therapeutic interventions addressing core GAD features (Stefan et al., 2019).

Concerning secondary outcomes, we indicated that NATs and dysfunctional attitudes decreased, whereas unconditional self-acceptance increased, after the CBT intervention, with comparable improvements in both groups. Previous research found that NATs are correlated with depression and anxiety, representing common characteristics among these disorders (Yapan et al., 2020). In concordance with our findings regarding the involvement of NATs in GAD, it was demonstrated that NATs were positively associated with specific symptoms of anxiety disorders (Iancu et al., 2015). Regarding the changes of dysfunctional attitudes following the CBT interventions, our research is aligned with Burns and Spangler (2001), who showed that dysfunctional attitudes were strongly correlated with anxiety and depression, at baseline and over the course of a 12-week CBT treatment. In the study cited above, post-treatment assessment also revealed a simultaneous reduction of anxiety, depression symptoms and dysfunctional attitudes (Burns and Spangler, 2001). In the same direction as our results, low unconditional self-acceptance proved to be an important predictor of negative affectivity (Popov, 2019). In contrast, high unconditional self-acceptance could function as a general protective factor for the development of psychopathology (Wild et al., 2017). Our research showed that the reduction of NATs and dysfunctional attitudes as well as the increase of unconditional self-acceptance are correlated to symptom changes after the treatment, which outlines the benefits of approaching GAD from a multilevel and comprehensive perspective.

Another important particularity of our study is represented by the fact that, to our knowledge, this is the first study that explored the feasibility of an extensive IM-VRCBT protocol for GAD. To date, the VRCBT (Rothbaum et al., 2006; Botella et al., 2007; Pitti et al., 2008; Robillard et al., 2010; Meyerbroeker et al., 2013; Triscari et al., 2015; Bouchard et al., 2017), leading to mixed conclusions. Similar to our study, a standard and a VR-augmented CBT protocol were associated with comparable results in terms of self-reports and behavioral measures (Anderson et al., 2013). Consequently, the outcomes from the IM-VRCBT group are concordant to other investigations indicating that exposure and relaxation VR scenarios are similarly effective to the classical, face-to-face version of these strategies in reducing GAD symptoms (Gorini and Riva, 2008; Meyerbröker and Emmelkamp, 2010; Guitard et al., 2019). We hypothesize that the combined CBT intervention we used, focusing on the cognitive, behavioral, and physiological functioning areas, may determine a prompt reduction of psychological distress after a brief treatment, despite that most protocols typically involve between 12 and 16 therapy sessions (Cuijpers et al., 2014; Robichaud et al., 2019). In addition, we discuss that using VR in psychotherapy could promote treatment adherence in GAD through the increased individual accessibility to this technology, which allows the emergence of self-guided programs (Anderson and Molloy, 2020). Besides, VR applications may improve the way exposure techniques are implemented by creating a highly standardized immersive environment, which could restrict the use of various safety strategies (e.g., avoiding to imagine feared scenarios).

Nevertheless, this study has some limitations. First, our sample consisted of a small number of participants selected from a young adult outpatient population. This does not allow the generalization of our outcomes to the entire population, neither the clear identification of change mechanisms in GAD. This aspect is also likely to affect the chance of finding a significant treatment x time interaction due to lower statistical power to detect such smaller improvements. Second, it was established that medical students develop more emotional disorders symptoms compared with the general population, especially during the preclinical years of medical school, due to the complexity of academic tasks and exams. It is possible that the anxiety levels observed in our samples could be more or less influenced by the context described above. Third, the investigation of two combined interventions that were compared simultaneously raises a major constraint due to the incapacity to indicate what specific processes counted for our results. This is particularly true regarding the efficiency of the VR-augmented intervention, since it is uncertain if the benefits of the treatment were correlated to the difference between psychological approaches, despite minor variations between the protocols, or the VR supplement. Therefore, an important direction for future studies would be to explore the advantages of VR-augmented CBT treatments for GAD, by comparing a stand-alone protocol with proved efficiency with the VR-enhanced version of the same intervention. Fourth, another drawback of our study is implied by the correlational analyses that do not permit the identification of potential causal effects. Fifth, this research did not include repeated measures for the assessment of symptom and process variables during the interventions. For this reason, the specific time when changes occurred and the interaction between primary and secondary measures could not be captured over the course of the CBT treatments. Sixth, our investigation did not include follow-up evaluations. Therefore, we are not able to draw firm conclusions about the differentiation between acute and chronic aspects of GAD. Also, the maintenance of the results on the long term could not be demonstrated.

## Conclusion

CBTs and IM-VRCBT are effective psychological interventions in the treatment of GAD. Even though both interventions are equally beneficial for the reduction of anxiety symptoms, the standard CBT was more effective for alleviating worries. Also, significant decreases in GAD-specific negative automatic thoughts and dysfunctional attitudes were associated with an increase in unconditional self-acceptance following both interventions. Furthermore, IM-CBT can be successfully augmented with VR in a single protocol for GAD treatment. Future studies could investigate the processes of change by incorporating multiple assessments of potential mediators throughout psychological treatments tailored for GAD.

## Data availability statement

The raw data supporting the conclusions of this article will be made available by the authors, without undue reservation.

## Ethics statement

The studies involving human participants were reviewed and approved by Ethics Committee of George Emil Palade University of Medicine, Pharmacy, Sciences and Technology of Targu Mures. The patients/participants provided their written informed consent to participate in this study.

## Author contributions

COP contributed to the development of intervention protocols, as well as the framework and procedure of the study. He also coordinated the training of the research team members and the process of data curation, wrote the first draft, and reviewed the manuscript. FAS led the statistical analysis, wrote the results section, and reviewed the manuscript. SM supervised the screening, was involved in the process of data curation, and wrote and reviewed the manuscript. AS was involved in the screening of participants, data curation, supervised the intervention process within the standard CBT group, wrote the first draft, and reviewed the manuscript. CMC was involved in the screening of participants, data curation, supervised the intervention process within the IM-VRCBT group, provided support for the first draft, and reviewed the manuscript. LMM was enrolled in the screening process, data curation, contributed to the first draft, and reviewed the manuscript. PO contributed to the design and framework of the study, coordination of the research project and feasibility, and ensured the allocation of funding and the availability of the equipment. All authors contributed to the article and approved the submitted version.

## Funding

This work was supported by the George Emil Palade University of Medicine, Pharmacy, Science and Technology of Targu-Mures, Research Grant number 239/7/14.01.2020.

## Conflict of interest

The authors declare that the research was conducted in the absence of any commercial or financial relationships that could be construed as a potential conflict of interest.

## Publisher’s note

All claims expressed in this article are solely those of the authors and do not necessarily represent those of their affiliated organizations, or those of the publisher, the editors and the reviewers. Any product that may be evaluated in this article, or claim that may be made by its manufacturer, is not guaranteed or endorsed by the publisher.
